# Identification of Host Biomarkers of Epstein-Barr Virus Latency IIb and Latency III

**DOI:** 10.1128/mBio.01006-19

**Published:** 2019-07-02

**Authors:** Joshua E. Messinger, Joanne Dai, Lyla J. Stanland, Alexander M. Price, Micah A. Luftig

**Affiliations:** a Department of Molecular Genetics and Microbiology, Duke Center for Virology, Duke University School of Medicine, Durham, North Carolina, USA; Johns Hopkins University; Johns Hopkins Bloomberg School of Public Health

**Keywords:** B lymphocyte, Epstein-Barr virus, gene expression, heterogeneity, lymphoma

## Abstract

Deciphering the molecular pathogenesis of virally induced cancers is challenging due, in part, to the heterogeneity of both viral gene expression and host gene expression. Epstein-Barr virus (EBV) is a ubiquitous herpesvirus prevalent in B-cell lymphomas of immune-suppressed individuals. EBV infection of primary human B cells leads to their immortalization into lymphoblastoid cell lines (LCLs), serving as a model of these lymphomas. In previous studies, reports from our laboratory have described a temporal model for immortalization with an initial phase characterized by expression of Epstein-Barr nuclear antigens (EBNAs), high levels of c-Myc activity, and hyperproliferation in the absence of the latent membrane proteins (LMPs), called latency IIb. This is followed by the long-term outgrowth of LCLs expressing the EBNAs along with the LMPs, particularly NFκB-activating LMP1, defining latency III. However, LCLs express a broad distribution of LMP1 such that a subset of these cells express LMP1 at levels similar to those seen in latency IIb, making it difficult to distinguish these two latency states. In this study, we performed mRNA sequencing (mRNA-Seq) on early EBV-infected latency IIb cells and latency III LCLs sorted by NFκB activity. We found that latency IIb transcriptomes clustered independently from latency III independently of NFκB. We identified and validated mRNAs defining these latency states. Indeed, we were able to distinguish latency IIb cells from LCLs expressing low levels of LMP1 using multiplex RNA-fluorescence *in situ* hybridization (RNA-FISH) targeting EBV *EBNA2* or *LMP1* and human *CCR7* or *MGST1*. This report defines latency IIb as a bona fide latency state independent from latency III and identifies biomarkers for understanding EBV-associated tumor heterogeneity.

## Abstract (precis)

EBV is a ubiquitous pathogen, with >95% of adults harboring a life-long latent infection in memory B cells. In immunocompromised individuals, latent EBV infection can result in lymphoma. The established expression profile of these lymphomas is latency III, which includes expression of all latency genes. However, single-cell analysis of EBV latent gene expression in these lymphomas suggests heterogeneity where most cells express the transcription factor, EBNA2, and only a fraction of the cells express membrane protein LMP1. Our work describes an early phase after infection where the EBNAs are expressed without LMP1, called latency IIb. However, LMP1 levels within latency III vary widely, making these states hard to discriminate. This may have important implications for therapeutic responses. It is crucial to distinguish these states to understand the molecular pathogenesis of these lymphomas. Ultimately, better tools to understand the heterogeneity of these cancers will support more-efficacious therapies in the future.

## INTRODUCTION

Epstein-Barr virus (EBV) is a large double-stranded DNA gammaherpesvirus that establishes life-long latent infection in resting memory B cells. Despite robust immune control in the vast majority of infected individuals, immunocompromised patients are at high risk for EBV-driven B-cell lymphomas. A model for these lymphomas is represented by EBV infection and immortalization of primary human B cells *in vitro* in lymphoblastoid cell lines (LCLs). Immortalized LCLs express all eight EBV latency proteins, consistent with latency III gene expression, including the EBV nuclear antigen (EBNA) transcription factors and the latent membrane proteins (LMPs), which are constitutively active receptor mimics (1–3).

However, EBV-infected B cells initially undergo a period of hyperproliferation characterized by expression of the EBNAs in the nearly complete absence of the LMPs, which is called latency IIb (4, 5). Early after infection, EBNA2 stimulates cellular proliferation by inducing the host transcription factor c-Myc through coordination of its upstream enhancer and chromatin looping (6). During this period, the cells are dependent upon MCL-1 and BCL-2 for survival in the absence of NFκB signaling (5, 7). Elevated levels of c-Myc early after infection antagonize LMP1 mRNA and protein expression (8). Low LMP1 levels early after infection may function to enable evasion of CD8^+^ T-cell recognition as LMP1-mediated NFκB activity promotes major histocompatibility complex class I (MHC-I) expression and peptide presentation (9, 10). By 2 to 3 weeks postinfection (wpi), c-Myc levels wane, hyperproliferation is attenuated, and full expression of the LMPs, particularly in the form of LMP1-mediated NFκB activity, is observed (11). These cells rely on NFκB signaling for survival (12) and display a distinct mitochondrial antiapoptotic phenotype with upregulation of BFL-1 (5, 7).

While latency III is characterized by full expression of the latent membrane proteins, it has long been observed that LMP1 levels vary widely within an LCL population. Flow cytometric analysis of LMP1 within bulk LCL populations shows an ∼100-fold range of LMP1 protein levels at single-cell resolution (13). This variable expression appears to be important for LCL homeostasis, as significantly elevated or depleted LMP1 levels result in reduced proliferation and cells sorted for high or low levels of LMP1 return to their full distribution within 18 h of sorting (10, 13). Therefore, levels of LMP1 expression within an LCL population fluctuate widely on a single-cell level and this wide distribution is important for LCL survival.

EBV is associated with several different lymphomas, including Hodgkin’s lymphoma, Burkitt lymphoma, and posttransplant lymphoproliferative disease (PTLD). However, the levels of viral latency gene expression in EBV-associated diseases are typically very heterogeneous. To understand the latency gene expression pattern in these diseases, immunohistochemical staining is employed to analyze the expression of LMP1 and EBNA2 in patient biopsy samples. Staining patient biopsy samples has demonstrated heterogeneity at the single-cell level where many cells may be positive for EBNA2 (EBNA2^+^) but negative for LMP1 (LMP1^–^) (14, 15). These cells are often quite common, as recent studies in a mouse model of coinfections with EBV and Kaposi’s sarcoma herpesvirus (KSHV) also identified a high frequency of EBNA2^+^/LMP1^−^ cells (16). Due to the wide distribution of LMP1 expression within a latency III LCL population, this technique does not enable distinguishing LMP1 low-latency III LCLs from LMP1 low-latency IIb cells.

We have previously demonstrated that latency IIb cells and latency III cells have unique survival requirements and forms of apoptotic regulation (7, 17, 18). However, those studies analyzed bulk LCL populations and did not address differences at the single-cell level. In this study, we addressed such single-cell heterogeneity by fluorescence-activated cell sorter (FACS) analysis of latency III LCLs based on the activity of surface ICAM-1 as a proxy for LMP1-mediated NFκB activity. Using this sorting strategy, we explored whether latency IIb cells are unique with respect to a subset of LCLs with low levels of NFκB that express reduced levels of LMP1. We also identified host transcriptomic markers of these latency states that are expressed in a latency stage-dependent manner but independently of LMP1 expression levels. Taking the results together, this work characterized latency IIb as a unique B-cell latency state of EBV infection and identified biomarkers that enable discrimination of latency IIb from latency III.

## RESULTS

### ICAM-1 is a proxy for LMP1-mediated NFκB target gene activation.

**FIG 1:**
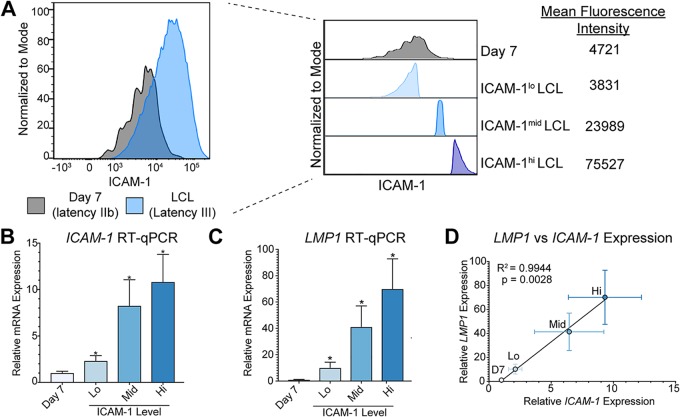
Using ICAM-1 as a proxy for LMP1 expression and LMP1-mediated NFκB signaling. (A) FACS analysis of ICAM-1 as a proxy for LMP1-mediated NFκB activity of 7 dpi EBV-infected latency IIb proliferating B cells and donor matched LCLs. The plot at the right shows sorting groups of latency IIb as well as the bottom, middle, and upper 15% of ICAM-1-expressing LCLs with corresponding MFI values. (B) RT-qPCR for ICAM-1 in sorted groups. Each bar represents the average of six independent matched donors. (C) RT-qPCR for LMP1 in sorted groups. Each bar represents the average of results from six independent matched donors. (D) Correlation between LMP1 RT-qPCR expression and ICAM-1 RT-qPCR expression. *, *P* < 0.05; **, *P* < 0.01; ***, *P* < 0.001 (by Student’s pairwise *t* test). All error bars denote standard errors of the means (SEM).

We have previously demonstrated that LMP1 levels are significantly lower early after EBV infection in primary human B cells than in immortalized LCLs (5). However, it is also known that LMP1 levels differ widely within an LCL population (10, 13). Due to the wide distribution of LMP1 expression levels in an LCL population, we first asked how these levels compare to those seen with early infected proliferating latency IIb cells. To assay this at the protein level, we conducted FACS analysis on early proliferating latency IIb cells (day 7 postinfection) and donor matched LCLs (>35 days postinfection) for ICAM-1 as a proxy for LMP1-mediated NFκB activity (19). As described for Fig. 1A, a subset of LCLs displayed low levels of NFκB activity similar to those seen with latency IIb cells, corroborating our previous data. Therefore, we concluded that these cells likely express comparable levels of LMP1 mRNA. However, we wondered whether these LMP1^lo^ LCLs were unique and thus different from latency IIb cells or whether they were cells that were “stuck” in latency IIb. To test this, we sorted latency IIb cells with respect to purity as well as the bottom, middle, and upper 15% fractions of ICAM-1-expressing cells within donor matched LCL populations (Fig. 1A). We used quantitative reverse transcription-PCR (RT-qPCR) analysis to validate that ICAM-1 levels were similar between latency IIb cells and ICAM-1^lo^ LCLs and that ICAM-1 mRNA abundance increased with increasing ICAM-1 mean fluorescence intensity (MFI) (Fig. 1B). Importantly, LMP1 mRNA abundance followed the same pattern as ICAM-1 (Fig. 1C). In fact, there was a direct linear correlation between ICAM-1 and LMP1 mRNA abundance, thereby validating our sorting strategy (Fig. 1D).

### Generation and validation of RNA-Seq libraries from EBV early infected and LCL populations sorted on the ICAM-1 level.

**FIG 2:**
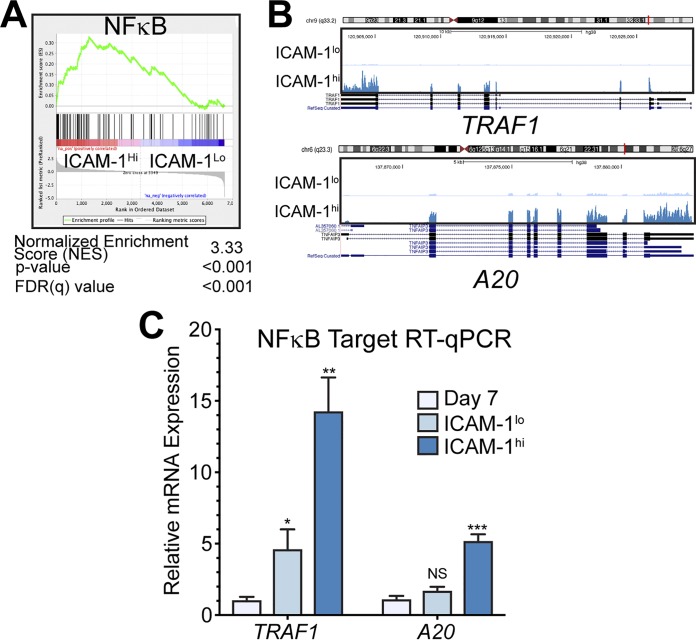
LCL populations are homogeneous despite a wide LMP1/NFκB distribution. (A) Motif preranked GSEA of differences between ICAM-1^lo^-expressing and ICAM-1^hi^-expressing LCLs. (B) RNA-Seq coverage map of known NFκB targets TRAF1 and A20 for ICAM-1^lo^ and ICAM-1^hi^ LCLs. (C) RT-qPCR analysis of TRAF1 and A20 in donor matched 7 dpi EBV-infected proliferating latency IIb cells and ICAM-1^lo^ or ICAM-1^hi^ LCLs. All RNA-seq coverage maps are on a 0-to-1,000 scale. Each bar represents the average of results from six independent matched donors. *, *P* < 0.05; **, *P* < 0.01; ***, *P* < 0.001 (by Student’s pairwise *t* test). All error bars denote SEM.

We next generated RNA sequencing (RNA-Seq) libraries to assess global gene expression differences between the following four populations: B cells at 7 days post-EBV infection (latency IIb) and LCLs with low, middle, or high levels of ICAM-1 (Fig. 1A). We first sought to validate our RNA-Seq libraries by querying the global gene expression differences between ICAM-1^lo^ and ICAM-1^hi^ LCLs. Consistent with our initial sorting and RT-qPCR experiments, we found by gene set enrichment analysis (GSEA) (20, 21) a significant enrichment in NFκB targets in ICAM-1^hi^ LCLs relative to ICAM-1^lo^ LCLs (Fig. 2A). Indeed, RNA-Seq coverage maps indicate that two well-described NFκB targets, TRAF1 and A20, are expressed at higher levels in ICAM-1^hi^ LCLs than in ICAM-1^lo^ LCLs (Fig. 2B). RT-qPCR analysis confirmed the RNA-Seq results (Fig. 2C), and these data suggest internal validation of both our sorting approach and our RNA-Seq pipeline.

### Host genes that distinguish EBV latency IIb cells from ICAM-1^lo^ LCLs are associated with DNA replication.

**FIG 3:**
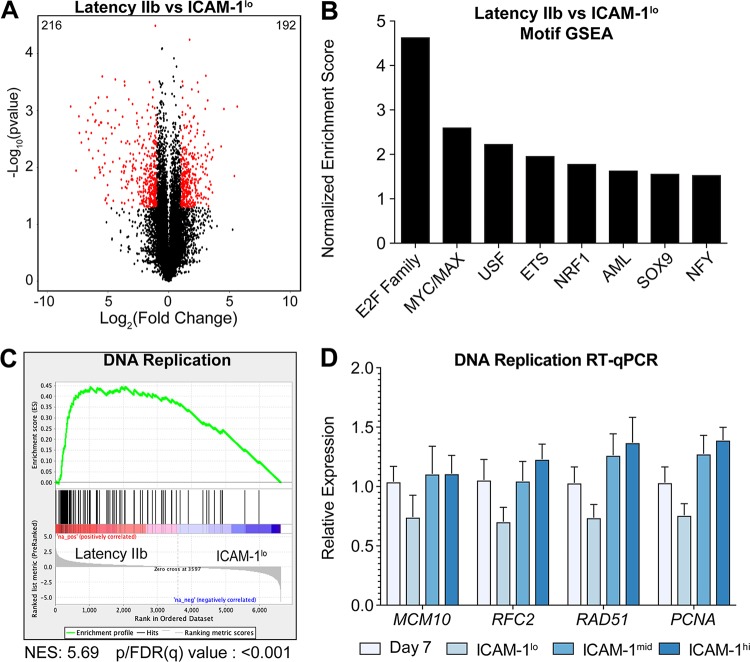
Latency IIb is defined by hyperproliferation and enhanced DNA replication. (A) Volcano plot of gene expression between latency IIb cells and ICAM-1^lo^ LCLs. Significantly regulated genes are indicated with red dots, and the associated data have *P* values of <0.05 and a log_2_(fold change) level of greater than 1 or less than negative 1. The number at top left indicates the number of significantly regulated genes in latency IIb, and the number at top right denotes the number of significantly regulated genes for ICAM-1^lo^. (B) Preranked motif GSEA results from comparisons between latency IIb cells and ICAM-1^lo^ LCLs. (C) Preranked GSEA gene ontology results from comparisons between latency IIb cells and ICAM-1^lo^ LCLs for DNA replication. NES, normalized enrichment score. (D) RT-qPCR between latency IIb cells and ICAM-1^lo^ LCLs for DNA replication genes. Each bar represents the average of results from six independent donors. Numbers above the bars indicate the number of times this motif was listed by GSEA. *, *P* < 0.05; **, *P* < 0.01; ***, *P* < 0.001 (by Student’s pairwise *t* test). All error bars denote SEM.

Our major goal in this study is to define the host genes that distinguish early infected latency IIb cells from ICAM-1^lo^ LCLs. Therefore, we performed a direct comparison of the genes differentially expressed between these two populations and identified 192 genes that were upregulated in the transition from early infected latency IIb cells to ICAM-1^lo^ LCLs and 216 genes that were downregulated from latency IIb cells to ICAM-1^lo^ LCLs (Fig. 3A; see also Table S1 and S2 in the supplemental material). We performed GSEA for transcription factor motifs upstream of the differentially expressed genes and found that E2F family transcription factors as well as MYC/MAX transcription factors were significantly enriched in latency IIb cells compared to ICAM-1^lo^ LCLs (Fig. 3B). GSEA also identified several gene ontology groups associated with DNA replication and mitotic division as the hallmark of latency IIb cells compared to ICAM-1^lo^ LCLs (Fig. 3C shows a representative plot). To validate these findings, we interrogated the expression levels of the genes associated with DNA replication by RT-qPCR. We found that MCM10, RFC2, RAD51, and PCNA were consistently upregulated but not significantly upregulated in latency IIb cells compared to ICAM-1^lo^ LCLs (Fig. 3D). Furthermore, this difference was not observed between latency IIb cells and ICAM-1^mid^ or ICAM-1^hi^ LCLs. Given our inability to identify host markers that distinguish latency IIb from latency III in members of this gene ontology group, we next sought to query the RNA-Seq data more broadly to identify such markers.

10.1128/mBio.01006-19.1 TABLE S1 List of genes downregulated from latency IIb to ICAM-1^lo^ LCL. Download Table S1, PDF file, 0.04 MB. Copyright © 2019 Messinger et al. 2019 Messinger et al. This content is distributed under the terms of the Creative Commons Attribution 4.0 International license.

10.1128/mBio.01006-19.2 TABLE S2 List of genes upregulated from latency IIb to ICAM-1^lo^ LCL. Download Table S2, PDF file, 0.03 MB. Copyright © 2019 Messinger et al. 2019 Messinger et al. This content is distributed under the terms of the Creative Commons Attribution 4.0 International license.

### EBV early infected latency IIb cells are transcriptomically distinct from latency III LCLs irrespective of ICAM-1/LMP1 levels.

**FIG 4:**
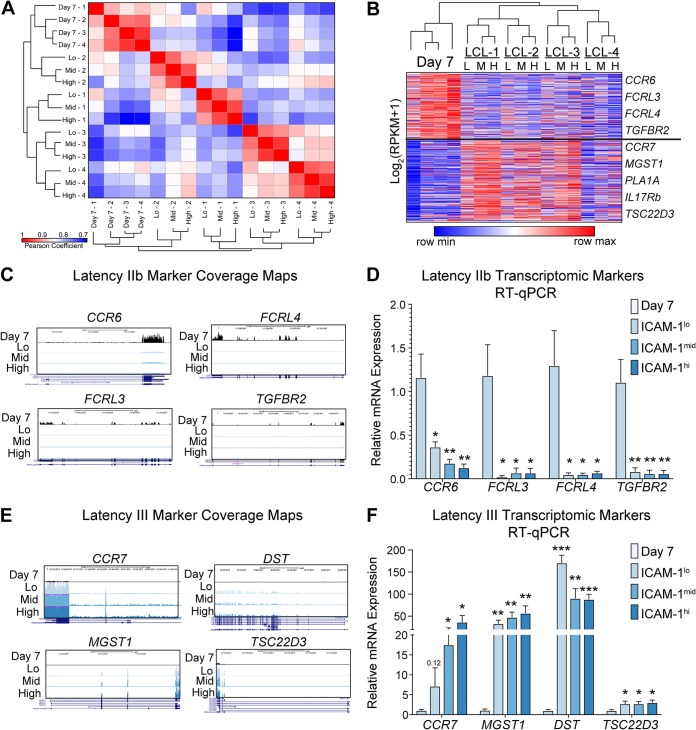
Latency IIb clusters uniquely from latency III independently of the donor, and each state contains transcriptionally unique host markers. (A) Pearson coefficient similarity matrix and hierarchical clustering between all samples used for RNA-Seq. (B) Gene expression heat map with hierarchical and k-means clustering of gene expression data from RNA-Seq. (C) RNA-Seq coverage maps for identified latency IIb-specific genes. (D) RT-qPCR validation of latency IIb-specific genes. (E) RNA-Seq coverage maps for identified latency III-specific genes. (F) RT-qPCR validation of latency III-specific genes. All RNA-seq coverage maps are on a scale of 0 to 300. Each bar represents the average of results from six independent matched donors. *, *P* < 0.05; **, *P* < 0.01; ***, *P* < 0.001 (by Student’s pairwise *t* test). All error bars denote SEM.

We next sought to assess whether the transcriptome of latency IIb cells differed significantly from that of ICAM-1-sorted LCL populations. We first generated a Pearson coefficient similarity matrix comparing the expression profiles of all 16 samples. Day 7 latency IIb transcriptomic profiles clustered together independently of donor and the ICAM-1/LMP1 expression level of the donor (Fig. 4A). The remaining clusters were comprised of the ICAM-1^lo^, ICAM-1^mid^, and ICAM-1^hi^ groups, with each donor clustering independently from the others. These results were further substantiated by unsupervised hierarchical clustering of the samples, where we found that day 7 latency IIb cells clustered independently of latency III LCLs (Fig. 4B). Therefore, gene expression differences between latency IIb cells and latency III cells are greater than those between donors and also between latency IIb cells and any ICAM-1-sorted population.

K-means clustering of the gene expression data generated profiles uniquely associated with latency IIb and latency III. Within these profiles, we identified significantly differentially expressed genes based on a greater than 2-fold change and *P* values of <0.05 in at least two of the three comparisons (day 7 versus ICAM-1^lo^, day 7 versus ICAM-1^mid^, and day 7 versus ICAM-1^hi^ LCLs). This analysis yielded 181 latency IIb-specific and 282 latency III-specific genes (Table S3). We chose four genes from each group with binary expression-like behavior to validate their specificity to latency IIb or latency III. Host biomarkers of latency IIb were *CCR6*, *FCRL3*, *FCRL4*, and *TGFBR2*. RNA-Seq coverage maps illustrated and RT-qPCR experiments validated the IIb specificity of these genes (Fig. 4C and D). Latency III biomarkers were *CCR7*, *MGST1*, *DST*, and *TSC22D3*, and these displayed similar binary gene expression-like behavior (Fig. 4E and F).

10.1128/mBio.01006-19.3 TABLE S3 List of latency IIb and latency III-specific genes. Download Table S3, PDF file, 0.03 MB. Copyright © 2019 Messinger et al. 2019 Messinger et al. This content is distributed under the terms of the Creative Commons Attribution 4.0 International license.

### Analysis of CCR6 and CCR7 surface expression as markers of latency IIb and III, respectively.

**FIG 5:**
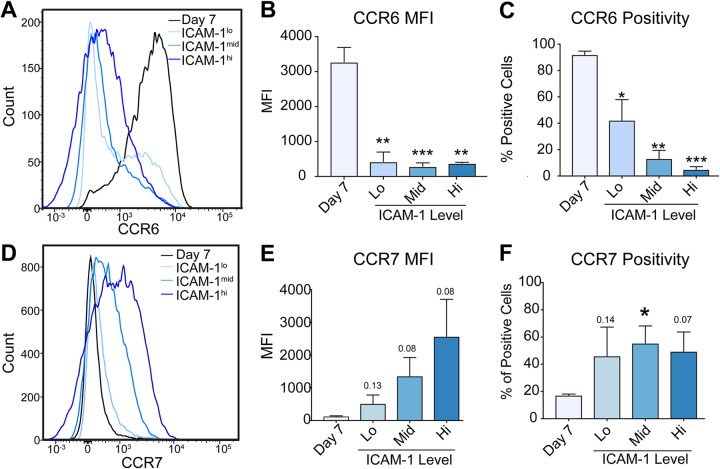
Protein validation of transcriptional markers reveals nonbinary latency specificity. (A) FACS analysis of CCR6 in day 7 latency IIb proliferating B cells and latency III LCLs stratified by ICAM-1 expression. (B) Quantification of mean fluorescence intensity of CCR6 expression data from panel A. (C) Percentage of CCR6-positive cells represented in panel A. (D) FACS analysis of CCR7 in day 7 latency IIb proliferating B cells and latency III LCLs stratified by ICAM-1 expression. (E) Quantification of mean fluorescence intensity of CCR7 expression presented in panel D. (F) Percentage of CCR7-positive cells represented in panel D. Each bar represents the average of results from 3 independent donors *, *P* < 0.05; **, *P* < 0.01; ***, *P* < 0.001 (by Student’s pairwise *t* test). All error bars denote SEM.

CCR6 and CCR7 displayed the strongest expression differences between latency IIb and latency III by RNA-Seq (Fig. 4C and E). As both of these proteins are surface expressed, we chose to investigate their utility as protein biomarkers to distinguish latency IIb from latency III. Flow cytometry of CCR6 indicated a strong downregulation of surface expression in comparing day 7 postinfection data to LCLs irrespective of the ICAM-1 level, corroborating our RNA-Seq and qRT-PCR data (Fig. 5A). While the MFI for CCR6 decreased significantly from the day 7 level to the levels seen with the LCLs (Fig. 5B), the difference in the percentages of positive cells dropped by only half between the day 7 level and the level seen with ICAM-1^lo^ LCLs (Fig. 5C). Similarly, while surface expression of CCR7 increased from the day 7 level to that seen with the LCL (Fig. 5D and E), the percentage of positive-cell results increased only about 2-fold (Fig. 5F). These data suggest that CCR6 and CCR7 protein levels will not suffice to distinguish between latency IIb-expressing and latency III-expressing cells.

### Multiplex RNA-FISH can distinguish latency IIb from latency III.

**FIG 6:**
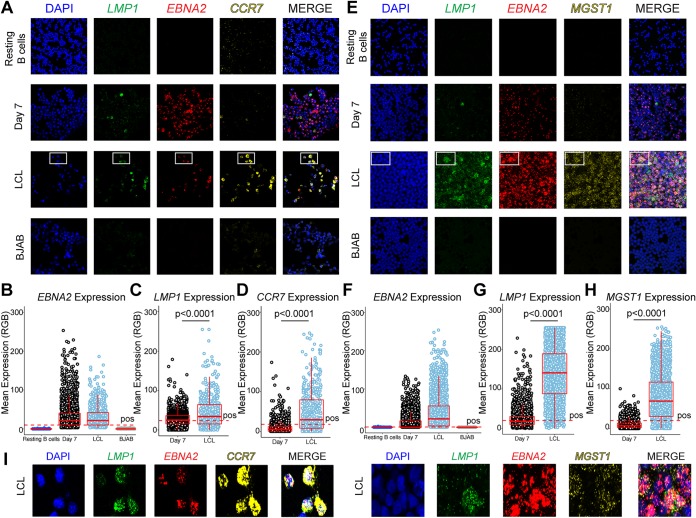
Multiplex RNA-FISH to distinguish EBV latency states. (A) Confocal immunofluorescence images from resting B cells, 7 dpi EBV^+^ latency IIb proliferating B cells, LCLs, and BJAB cells stained with LMP1, EBNA2, and CCR7 RNA FISH probes. (B) Quantification of the EBNA2 expression data presented in panel A. RGB, red green blue. (C) Quantification of data representing LMP1 expression in EBNA2^+^ cells presented in panel A. (D) Quantification of data representing CCR7 expression in EBNA2^+^ cells presented in panel A. (E) Analyses were performed as described for panel A but for LMP1, EBNA2, and MGST1 mRNA. (F) Quantification of EBNA2 expression data presented in panel E. (G) Quantification of data representing LMP1 expression in EBNA2^+^ cells presented in panel E. (H) Quantification of data representing MGST1 expression in EBNA2^+^ cells presented in panel E. (I) Magnification of boxed region in LCLs shown in panels A and E for EBNA2, LMP1, and CCR7 or MGST1 expression. For all quantifications, data representing day 7 cells and LCLs are averages of results from 3 independent donors and 7 fields of view per donor. For resting B cells, the data are representative of one blood donor and 7 fields of view. BJAB data also represent averages from analyses of 7 fields of view. *, *P* < 0.05; **, *P* < 0.01; ***, *P* < 0.001 (by one-tailed Mann-Whitney nonparametric *t* test). All error bars denote SEM.

Given the challenges of protein-based biomarker validation, we sought to use multiplex RNA fluorescence *in situ* hybridization (RNA-FISH) to leverage our RNA-based biomarker discovery approach. As detailed in Fig. 4E, the mRNA expression level of *CCR7* correlated with latency III independently of ICAM-1/LMP1 levels. CCR7 was also the most highly expressed latency III-specific mRNA. Therefore, we designed probes to detect CCR7 mRNA along with EBNA2 and LMP1 mRNAs. Our hypothesis predicted that latency IIb (EBNA2^+^/LMP1^−^) cells would be CCR7 negative and that latency III cells would be CCR7 positive irrespective of the LMP1 level. We tested this hypothesis in sorted, proliferating day 7 (latency IIb) infected cells and LCLs with resting B cells and the EBV-negative B-lymphoma BJAB cell line as negative controls (Fig. 6A). EBNA2 expression was robust in EBV-infected day 7 cells and LCLs compared to resting B cells and BJAB cells (Fig. 6A and B). LMP1 expression was low in the day 7 cells and higher (but heterogeneous) in the LCLs, as expected (Fig. 6A to C). CCR7 levels were low in EBNA2^+^ day 7 cells and significantly higher in LCLs independently of LMP1 level (Fig. 6D).

While CCR7 was the most highly expressed latency III-specific transcript, MGST1 displayed a higher fold change between day 7 cells and LCLs and low donor-to-donor variation. Therefore, we tested the ability of MGST1 to distinguish latency IIb from latency III by RNA-FISH (Fig. 6E). Again, we found that the levels of EBNA2 mRNA expression in latency IIb and latency III displayed similar distributions and that the level of LMP1 mRNA increased from latency IIb to latency III, as expected (Fig. 6F and G). Importantly, MGST1 mRNA levels significantly increased from latency IIb to latency III, demonstrating that MGST1 is a host-specific marker of latency III (Fig. 6H). Finally, both CCR7 and MGST1 were capable of distinguishing LMP1^lo^ LCLs from latency IIb cells. Indeed, the full heterogeneity of LMP1 expression levels in latency III is visualized in Fig. 6I, where all cells are shown to be EBNA2 positive and CCR7 or MGST1 positive. Thus, both CCR7 mRNA and MGST1 mRNA are reliable host transcriptomic markers of EBV latency III.

## DISCUSSION

In this study, we identified host biomarkers that distinguish EBV latency IIb from latency III. We recently established that the initial infection of primary human B cells with EBV displays a latency IIb phenotype where the viral EBNA proteins are expressed in the nearly complete absence of the LMPs (5, 22). However, latency III is observed later during primary infection where the EBNAs and LMPs are all expressed as seen in LCLs. The broad distribution of LMP1 within latency III populations makes these cells difficult to distinguish. As many EBV-positive tumors display cellular LMP1 heterogeneity (14, 15), it is important to determine whether these EBNA^+^/LMP1^−^ cells are latency IIb or latency III, as their immune recognition and response to chemotherapy may vary depending on viral gene expression levels (7, 10).

To address this issue, we first confirmed that the NFκB-induced surface protein ICAM-1 is a proxy and reporter for LMP1-mediated NFκB signaling and LMP1 mRNA levels (19). We confirmed the broad range of LMP1/NFκB expression in latency III as observed by others (10, 13) and found a significant overlap of ICAM-1 surface levels in the latency IIb early infected cells and ICAM-1^lo^ LCLs. Through FACS analysis coupled with RNA-Seq, we found that the major determinant of the differences between LMP1^lo^-expressing and LMP1^hi^-expressing LCLs is indeed NFκB signaling, with a small component of cell cycle regulation through E2Fs. Importantly, we found that latency IIb gene expression profiles clustered with characteristics distinct from those seen with latency III irrespective of the LMP1 level or donor from which they were generated. Thus, latency IIb is a bona fide latency state.

We identified and validated four latency IIb-specific and four latency III-specific host mRNAs that were differentially regulated between the states. CCR6, FcRL4, FcRL3, and TGFBR2 were specific to latency IIb, while CCR7, MGST1, DST, and TSC22D3 were specific to latency III. Interestingly, *CCR7* was one of the first genes demonstrated to be induced by EBV, originally being called EBI1 (23). However, surface expression of CCR6 and CCR7 did not fully distinguish latency IIb from latency III. For this reason, we turned to multiplexed RNA-FISH to simultaneously measure viral and host mRNA levels in single cells. This approach enabled us to identify CCR7 and MGST1 as host biomarkers of latency III independently of LMP1 level.

While protein-based expression analysis by immunohistochemistry (IHC) is the gold standard in pathology laboratories, multiplex RNA-FISH is a promising new approach to decipher cellular heterogeneity in tumors (24, 25). This approach is as sensitive as IHC but lacks its limitations with respect to antibody specificity and sensitivity (26, 27). Indeed, multiplex RNA-FISH overcomes the issue of protein epitope variation or antigen retrieval by tiling probes for the target gene across the entire length of the mRNA. In our studies, multiplexing probes with distinct fluorophores enabled the detection of both viral mRNAs and host mRNAs at single-cell resolution.

EBV-positive lymphomas in immune-suppressed patients have been characterized to display latency III gene expression (EBNA^+^/LMP^+^) (22). However, several early pathology studies of EBV-positive PTLD and HIV lymphomas as well as more recent mouse models described a significant EBNA^+^/LMP^−^ cell population (14, 15). In a cord blood mouse model of EBV infection, EBNA2^+^/LMP1^−^ cells were observed at a high frequency whereas latency III cells were rarely detected. This was hypothesized to be due to the increased immunogenicity of latency III cells (28). Similarly, in a recent study using a mouse model of EBV/KSHV coinfection, latency IIb cells were detected at a high frequency (16). The pathophysiological relevance of latency IIb therefore supports the results of our study with respect to defining latency-distinguishing host markers.

Autologous and allogeneic T-cell therapies targeting MHC-restricted viral antigens are used in the treatment of EBV-associated PTLD (29–35). These products are typically highly enriched for CD8^+^ cytotoxic T cells; therefore, proper EBV antigen presentation through MHC class I within these tumors is likely important for a robust clinical response. As LMP1 expression in a latency III B cell cycles between subpopulations that are LMP1^hi^ and highly sensitive to CD8^+^ T-cell killing and those that are LMP1^lo^ and much less sensitive (10), it will be important to distinguish whether EBNA2^+^/LMP1^−^ cells within PTLD tumors are LMP1^lo^ latency IIb cells of fixed status or are latency III cells cycling between low and high LMP1 states. PTLD tumors with persistent latency IIb infection may be more difficult to treat with T-cell therapies than latency III-predominant PTLD. This remains to be tested by correlating EBV latency type in PTLD tissue with response to T-cell therapy.

Recent clinical studies have led to the development of LMP-specific cytotoxic T lymphocytes (CTLs) for the treatment of EBV latency IIa tumors (LMP^+^/EBNA^−^) (36–38). For EBV-associated PTLD with predominately latency III gene expression, LMP-specific CTLs would be expected to have clinical efficacy and, indeed, a clinical trial is under way (ClinicalTrials registration no. NCT02900976). In light of our findings regarding latency IIb, it remains pertinent to consider screening these tumors for LMP1 expression and perhaps excluding tumors that display a latency IIb expression phenotype. Coupling of the host biomarkers that we have identified with the viral EBNA2 and LMP1 using multiplex RNA-FISH could provide significant predictive power in screening these tumors for efficacy using T-cell therapies and chemotherapeutics.

## MATERIALS AND METHODS

### Cell lines, culture conditions, and viruses.

Peripheral blood mononuclear cells (PBMCs) were obtained from whole blood from the Gulf Coast Regional Blood Center (Houston, TX) via centrifugation over a Ficoll Histopaque-1077 gradient (Sigma, catalog no. H8889). The B95-8 strain of Epstein-Barr virus was generated from the B95-8 Z-HT cell line as previously described (39). Virus infections were performed by adding either 100 μl of filtered B95-8 Z-HT supernatant to 10^6^ PBMCs or 500 μl of B95-8 Z-HT per 10^6^ B cells, as determined by FACS analysis.

Cell lines were cultured in RPMI 1640 media supplemented with 10% (LCLs) or 15% (primary B cells) fetal bovine serum (FBS) (Corning), 2 mM l-glutamine (Invitrogen), 100 U/ml penicillin, and 100 μg/ml streptomycin (Invitrogen). All cells were maintained at 37°C in a humidified incubator with 5% CO_2_.

### Flow cytometry and sorting.

To track cellular division, cells were stained with CellTrace violet (Invitrogen, catalog no. C34557), a fluorescent proliferation-tracking dye. For analytical panels, 10^6^ PBMCs on day 7 postinfection with EBV B95-8 or 10^6^ LCLs were washed once with FACS buffer (phosphate-buffered saline [PBS] plus 5% FBS) and stained with the following antibodies (in isolation or in combination for 30 to 60 min in the dark at 4°C): ICAM-1 phycoerythrin (PE) (BioLegend, catalog no. 353106), CCR6 PE/Dazzle (BioLegend, catalog no. 353430), CCR7 PE/Dazzle (BioLegend, catalog no. 353236), and CD19 allophycocyanin (APC) (BioLegend, catalog no. 302212). Cells were washed once with FACS buffer after incubation, and 10,000 blank counting beads (Spherotech, catalog no. ACBP-50-10) were added to each tube. Data were collected on a BD LSRFortessa cell analyzer, and 1,000 blank beads were used as the stopping gate. All samples were stained and subjected to FACS analysis at the same time to ensure consistency in analysis. Marker positivity was determined using matched fluorescence minus one control.

For sorting experiments, proliferating cells were sorted to purity using CD19 APC (BioLegend, catalog no. 302212) positivity as well as a dilution of CellTrace violet (CD19^+^/CTV^lo^) on a MoFlo Astrios cell sorter at the Duke Cancer Institute Flow Cytometry Shared Resource. LCLs were sorted to purity after staining was performed with ICAM-1 PE (BioLegend, catalog no. 353106) and were gated for the bottom, middle, and upper 15% fractions of ICAM-1-expressing cells.

### RNA-Seq and analysis.

Whole RNAs from sorted early EBV-infected latency IIb B cells and from sorted donor matched LCLs were isolated using an RNeasy kit (Qiagen, catalog no. 74104). mRNA sequencing libraries were prepared using a Kappa stranded RNA-Seq library preparation kit (Kappa Biosystems, catalog no. KR0934) and sequenced on an Illumina Hiseq 4000 system at the Duke University Sequencing and Genomics Shared Core Facility. Resulting single-end, unpaired reads were aligned to the human genome (hg38) using Hisat2 (40). Resulting BAM files were converted to SAM files using samtools, and transcripts were assembled using Stringtie. Assembled transcripts were quantified using the R package ballgown. Normalized reads per kilobase per million (RPKM) values were exported from ballgown and used for heat map visualization and log_2_(RPKM+1) calculations. Statistical significance and false positivity were determined using ballgown. Heat maps were generated using Morpheus from the Broad Institute (https://software.broadinstitute.org/morpheus/), and similarity matrices were created using the R package pheatmap (https://CRAN.R-project.org/package=pheatmap). RNA-Seq coverage maps were generated using UCSC Genome Browser in a Box (GBiB) (41).

### RNA isolation, RT-qPCR, and primers used.

Total RNA from sorted EBV-infected early latency IIb proliferating B cells or sorted LCLs was isolated using an RNAeasy kit (Qiagen, catalog no. 74104) according to the manufacturer’s instructions. One microgram of total RNA was reverse transcribed into cDNA using a High-Capacity cDNA reverse transcription kit (Applied Biosystems, catalog no. 4368814) according to the manufacturer’s instructions. Resulting cDNA was diluted in ultrapure H_2_O, and 5 ng per reaction was used for RT-qPCR with the SYBR green (Quantabio, catalog no. 95054) detection method. Relative expression was calculated using the ΔΔ*C_T_* (threshold cycle) method with SETDB1 as an endogenous control. Table 1 in the supplemental material lists all primers used for RT-qPCR in this study.

**TABLE 1:** Primers used for RT-qPCR

Gene name	Forward primer (5′→3′)	Reverse primer (5′→3′)
ICAM-1	ATGCCCAGACATCTGTGTCC	GGGGTCTCTATGCCCAACAA
LMP1	AATTTGCACGGACAGGCATT	AAGGCCAAAAGCTGCCAGAT
TRAF1	TCCTGTGGAAGATCACCAATGT	GCAGGCACAACTTGTAGCC
A20	TTGTCCTCAGTTTCGGGAGAT	ACTTCTCGACACCAGTTGAGTT
CCR6	TTCAGCGATGTTTTCGACTCC	GCAATCGGTACAAATAGCCTGG
FCRL3	GTAAGAAGCCTGGGTAGAAAGAC	GCTGCACAGTAGTATCTCCCTG
FCRL4	TCTTCAGACTCCTTAATCCTG	CCAAGTATATTTCACAGCAGTC
TGFBR2	AAGATGACCGCTCTGACATCA	CTTATAGACCTCAGCAAAGCGAC
CCR7	ATTTGTGTGGGCCTACTG	TCATGGTCTTGAGCCTCTTGA
MGST1	ATGACAGAGTAGAACGTGTACGC	TACAGGAGGCCAATTCCAAGA
DST	CTACCAGCACTCGAACCAGTC	GCCGAAGCTAATGCAAGAGTTG
TSC22D3	AACACCGAAATGTATCAGACCC	TGTCCAGCTTAACGGAAACCA
MCM10	CCCCTACAGACGATTTCTCGG	CAGATGGGTTGAGTCGTTTCC
RAD51	CAACCCATTTCACGGTTAGAGC	TTCTTTGGCGCATAGGCAACA
PCNA	CCTGCTGGGATATTAGCTCCA	CAGCGGTAGGTGTCGAAGC
RFC2	GTGAGCAGGCTAGAGGTCTTT	TGAGTTCCAACATGGCATCTTTG
SETDB1	TCCATGGCATGCTGGAGCGG	GAGAGGGTTCTTGCCCCGGT

### RNA-FISH.

RNA-FISH was conducted using the Advanced Cell Diagnostics (ACD) RNA Scope multiplex fluorescent v2 assay (Advanced Cell Diagnostics, catalog no. 323100). In brief, resting B cells isolated from peripheral blood (BD IMAG human B lymphocyte enrichment set—DM, BD catalog no. 558007), were used in sorting latency IIb proliferating B cells on day 7 postinfection (dpi), and LCLs were washed once in PBS, fixed in 10% neutral buffered formalin for 1 h at 37°C, washed again in PBS, and resuspended in 70% ethanol (EtOH) before being cytospun onto glass slides using a Cyto-Tek Sakura table-top cytofuge at ∼735 × *g* for 22 min. Slides were dried for 20 min before being fixed in an ethanol gradient of 50%, 70%, and 100% EtOH for 5 min for each gradient step. Slides were stored overnight at −20°C in 100% EtOH before being dried and having a hydrophobic barrier applied to the slide using an ImmuEdge pen (Vector, catalog no. H-4000). Samples were first treated with peroxide for 10 min at room temperature (RT) to quench endogenous peroxidase. After peroxide treatment, we treated the cells with ACD protease III for 30 min at 40°C before proceeding to the standard RNA-SCOPE multiplex fluorescent V2 assay protocol, performed according to the manufacturer’s instructions. Cells were stained with a probe mixture containing HHV4-LMP1-C1 (Advanced Cell Diagnostics, catalog no. 414681), HHV4-EBNA2-C2 (Advanced Cell Diagnostics, catalog no. 547771-C2), and either Hs-CCR7-C3 (Advanced Cell Diagnostics, catalog no. 410721-C3) or Hs-MGST1-C3 (Advanced Cell Diagnostics, catalog no. 538721-C3). After hybridization, the signal was amplified and conjugated to fluorescein (Perkin Elmer, catalog no. NEL741E001KT), Cy3 (catalog no. NEL744001KT), or Cy5 (catalog no. NEL745001KT) TSA secondary antibody. Slides were stained with DAPI (4′,6-diamidino-2-phenylindole) before being mounted with ProLong Gold antifade (Invitrogen, catalog no. P10144). Slides were dried for 30 min at room temperature before being moved to 4°C for long-term storage. All images were acquired on a Zeiss 780 upright confocal microscope, and resulting images were analyzed with Fiji software.

### Fiji image analysis.

Images were processed using in-house Fiji macros. The macro performs the following functions. The DAPI image and corresponding fluorescent channel image are simultaneously imported into Fiji for each sample. A Gaussian blur (σ = 2) is applied to the DAPI image, and then an Otsu threshold is applied. The DAPI image is then converted to binary data, and the watershed function is then applied to distinguish potentially overlapping nuclei. A threshold is then applied to the fluorescent channel image (automatic for fluorescein and Cy5, minimum value for Cy3). The DAPI image is subsequently selected, and the Fiji Set Measurements window is utilized to report the area and mean, minimum, and maximum intensity data are redirected to the fluorescent channel image. Fiji Analyze particles are then used to determine the intensity of the foci in the fluorescent channel image that lie within the boundaries identified by the DAPI channel image.

Once the macro had been applied to all images for all fluorescent channels, all of the raw data were curated. The expression levels of day 7 cells and LCLs stained with positive-control and negative-control probes provided by the manufacturer (Advanced Cell Diagnostics, catalog no. 321801 and 321831, respectively) were plotted on a histogram, and positivity thresholds were set at the point where the positive-control and negative-control histograms intersected (data not shown). For EBNA2-Cy3, a minimum threshold is used that allows strict discrimination between EBV^+^ and EBV^−^ cells. For LMP1-fluorescein, CCR7-Cy5, or MGST1-Cy5, to allow greater tolerance of “low” levels of expression, a less strict “automatic” thresholding method was used.

With cutoff values for EBNA2 having been established, the data were subsequently curated to report LMP1 and CCR7 expression only in the cells that were positive for EBNA2 mRNA to ensure that we analyzed only EBV-infected cells. Due to the non-Gaussian distribution of the data corresponding to this positive signal, a Mann-Whitney nonparametric *t* test was used to determine statistical significance.

### Data availability.

The RNA-Seq data have been uploaded to the Gene Expression Omnibus (GEO) database under accession number GSE132138.
