# Supplementary material for: Identification of Host Biomarkers of Epstein-Barr Virus Latency IIb and Latency III
Source: mBio. 2019 Jul 2;10(4):e01006-19. doi: 10.1128/mBio.01006-19 (PMC6606803; doi:10.1128/mBio.01006-19)
Supplement: TABLE S1 [file mBio.01006-19-st001.pdf]

Supplamentary Table 1 - Genes Downregulated from Latency IIb to ICAM1lo

gene name	fc	pval	qval	log2FC
LYZ	0.03128	0.001014	0.194485	-4.998618
FCRL4	0.059179	0.001767	0.194485	-4.07876
FCRL3	0.066846	0.003527	0.209944	-3.903009
CD24	0.080857	0.042864	0.313285	-3.628486
PTPRC	0.09029	0.017566	0.26097	-3.469285
TGFBR2	0.095451	0.00346	0.209944	-3.389095
ZBED2	0.096699	0.019749	0.263611	-3.370354
FCER1G	0.10166	0.017775	0.261004	-3.298182
OSBPL10	0.102329	0.000634	0.194485	-3.288718
BZW2	0.118397	0.01634	0.255513	-3.078301
CPT1A	0.123162	0.015831	0.255239	-3.021366
ARL4C	0.123991	0.049927	0.324438	-3.011694
TRIB2	0.126551	0.009504	0.23416	-2.982207
RASGRP2	0.127007	0.000767	0.194485	-2.977014
GAPT	0.128086	0.016508	0.255513	-2.964812
STAC3	0.130522	0.010281	0.236821	-2.937633
TSPAN13	0.130799	0.008914	0.232034	-2.934574
FAM46C	0.133013	0.037497	0.303348	-2.910357
KLHL14	0.135304	0.007038	0.218509	-2.885725
RNF130	0.146112	0.003505	0.209944	-2.77485
RBMS1	0.150735	0.010822	0.23808	-2.72991
RASGEF1B	0.154975	0.007571	0.221649	-2.689896
C16orf74	0.161271	0.001106	0.194485	-2.632444
CHST15	0.162797	0.001717	0.194485	-2.61885
IMPA2	0.167273	0.015649	0.254853	-2.579724
SATB1	0.168358	0.003357	0.209944	-2.570398
PMEPA1	0.169679	0.018858	0.262359	-2.559123
SETD7	0.173483	0.027021	0.281608	-2.52713
S100A10	0.178445	0.001982	0.194485	-2.486448
LPAR6	0.188549	0.021801	0.268953	-2.406992
MT1E	0.189622	0.016501	0.255513	-2.398805
FOXP1	0.202058	0.032481	0.29065	-2.307155
HIP1R	0.20406	0.010193	0.236821	-2.292936
SERPINB6	0.208431	0.010173	0.236821	-2.262356
FCRL2	0.208822	0.027005	0.281608	-2.259654
PDE3B	0.213355	0.01531	0.253597	-2.228672
SGALNACT	0.213625	0.03828	0.30563	-2.226847
JADE3	0.215656	0.0432	0.314289	-2.213196
CR1	0.216457	0.042997	0.313377	-2.20785
TGIF1	0.216867	0.004562	0.213018	-2.205116
CCR6	0.219125	0.001325	0.194485	-2.190174

ADD2	0.223499	0.004237	0.213018	-2.161661
JAM3	0.228117	0.042589	0.313285	-2.132153
HHEX	0.232253	0.027367	0.282024	-2.106232
CCL25	0.233	0.037318	0.302708	-2.101597
GRAP	0.23437	0.032063	0.289106	-2.093138
FAM177B	0.23495	0.026284	0.279989	-2.089571
SERPINB1	0.235423	0.021338	0.267827	-2.086671
GNB4	0.236638	0.039429	0.309156	-2.079249
UGT8	0.240308	0.018459	0.262168	-2.057046
ST14	0.241647	0.031401	0.287819	-2.049026
CXCL10	0.241673	0.045566	0.317946	-2.04887
MYO1F	0.242292	0.035506	0.297014	-2.045181
PLEKHG1	0.247142	0.010724	0.237567	-2.016587
WASF1	0.249245	0.048033	0.32202	-2.004363
TBC1D9	0.259214	0.018469	0.262168	-1.947786
TRPM2	0.259317	0.026143	0.279447	-1.947212
NUGGC	0.26272	0.039924	0.309789	-1.928404
GALNT3	0.262853	0.025029	0.276966	-1.927671
CD83	0.263645	0.0162	0.255513	-1.923329
ANKLE1	0.266542	0.010881	0.238315	-1.907564
LINGO3	0.266828	0.026664	0.279989	-1.906017
CAPN2	0.267809	0.019723	0.263611	-1.900725
SLA2	0.268449	0.013064	0.249142	-1.89728
STMN1	0.275411	0.009429	0.233908	-1.860342
SERPINA9	0.276346	0.017409	0.26097	-1.855454
PM20D2	0.276579	0.048716	0.323235	-1.854237
VPS37B	0.278026	0.033901	0.293505	-1.846708
MED13L	0.280717	0.035499	0.297014	-1.832812
IMPACT	0.283304	0.011486	0.242207	-1.819577
OGFRL1	0.285939	0.019902	0.263727	-1.80622
SCML1	0.288254	0.017734	0.261004	-1.794589
GBP1	0.288918	0.047191	0.320843	-1.791269
KCNK6	0.291345	0.00615	0.218509	-1.7792
CPM	0.295097	0.000989	0.194485	-1.76074
SPOCK2	0.295913	0.010433	0.236821	-1.756755
NMRAL1	0.299487	0.028127	0.284251	-1.739434
NMRAL1	0.299487	0.028127	0.284251	-1.739434
RNASE1	0.300509	0.049268	0.32395	-1.73452
MCM10	0.303443	0.015224	0.253597	-1.720503
MOB3B	0.309811	0.034803	0.294716	-1.690538
ZNF860	0.3112	0.001722	0.194485	-1.684086
ZNF486	0.31427	0.005225	0.216866	-1.669925
CDC6	0.314374	0.024224	0.274864	-1.669446
CXCL9	0.318267	0.027015	0.281608	-1.651689
DOCK9	0.320266	0.0475	0.321108	-1.642658

BACH2	0.322507	0.022491	0.271277	-1.632599
KCNC3	0.324044	0.017832	0.261004	-1.625741
CLSPN	0.326793	0.021867	0.269259	-1.61355
HPSE	0.329551	0.036117	0.298141	-1.601428
COQ2	0.3301	0.042578	0.313285	-1.599025
DOK2	0.331366	0.041079	0.311117	-1.593504
JAZF1	0.33233	0.013391	0.249142	-1.589313
HENMT1	0.336588	0.024882	0.276966	-1.570944
TAF1A	0.337145	0.044128	0.31587	-1.568561
ZSCAN18	0.339892	0.030009	0.286166	-1.556851
TRIP13	0.341556	0.010566	0.236821	-1.549804
MMP9	0.342442	0.035258	0.296243	-1.546068
GPD1L	0.342815	0.022653	0.271705	-1.544499
CDC45	0.343013	0.031103	0.287819	-1.543666
KDM7A	0.343387	0.032556	0.290693	-1.542092
MTSS1	0.344045	0.035277	0.296243	-1.53933
MCM4	0.345548	0.044088	0.31587	-1.53304
RAVER2	0.34796	0.009103	0.232624	-1.523006
PWWP2B	0.348212	0.028744	0.284251	-1.521963
DDX60L	0.350127	0.049548	0.32395	-1.514048
LDOC1	0.35185	0.031229	0.287819	-1.506967
FAM81A	0.355605	0.035549	0.297107	-1.491652
APOBR	0.356306	0.040599	0.310749	-1.488813
DERL3	0.356313	0.014116	0.250004	-1.488782
ELL3	0.356418	0.001326	0.194485	-1.488357
DHFR	0.357309	0.00283	0.207585	-1.484754
FAM111B	0.359114	0.006895	0.218509	-1.477484
RAD51	0.361583	0.015443	0.254526	-1.467603
GBP1P1	0.363706	0.039098	0.308177	-1.459154
POLE2	0.365358	0.010496	0.236821	-1.452617
TYROBP	0.368582	0.017236	0.260238	-1.439943
TIMELESS	0.369769	0.0233	0.273049	-1.435304
PTPRO	0.369931	0.027694	0.282854	-1.43467
ARHGAP42	0.371944	0.035339	0.296509	-1.426843
DOPEY2	0.372087	0.009351	0.233624	-1.426289
GINS2	0.372626	0.040272	0.310384	-1.424199
REL	0.372923	0.029037	0.284254	-1.423049
DSCC1	0.373355	0.012337	0.246152	-1.421381
NDFIP1	0.379433	0.021068	0.267819	-1.398083
ESCO2	0.390623	0.005832	0.216866	-1.35615
DTL	0.390628	0.026575	0.279989	-1.356132
IFNLR1	0.391351	0.029611	0.285409	-1.353466
LFNG	0.394743	0.011975	0.244367	-1.341013
ALDH16A1	0.397742	0.038852	0.30773	-1.330094
OSBPL8	0.398219	0.000852	0.194485	-1.328367

C1orf220	0.401286	0.000585	0.194485	-1.317296
HEBP1	0.402577	0.015508	0.254526	-1.312665
RASA2	0.40305	0.025404	0.277835	-1.310971
MCM6	0.406024	0.016047	0.255513	-1.300363
PPP3CA	0.408701	0.013801	0.249142	-1.290884
WHAMMP1	0.409585	0.003507	0.209944	-1.287764
ZWINT	0.411443	0.048624	0.323155	-1.281234
BRCA1	0.414443	0.000427	0.194485	-1.270754
IFI27L1	0.41484	0.020511	0.265408	-1.269372
IFI27L1	0.41484	0.020511	0.265408	-1.269372
GSG2	0.416787	0.016396	0.255513	-1.262619
ZNF682	0.418647	0.030857	0.287819	-1.256194
NUDT1	0.419067	0.045636	0.318128	-1.254746
GINS4	0.421105	0.022555	0.271582	-1.24775
CLCF1	0.421559	0.041425	0.311902	-1.246195
MLXIP	0.422159	0.029391	0.284754	-1.244143
MPEG1	0.42231	0.016255	0.255513	-1.243627
RBL1	0.423259	0.004582	0.213018	-1.240386
MND1	0.423303	0.006331	0.218509	-1.240238
SKA3	0.424108	0.009744	0.235389	-1.237497
SIGIRR	0.429094	0.007665	0.222324	-1.220634
ORC1	0.429623	0.04789	0.32202	-1.218857
ADAM15	0.432582	0.033016	0.291815	-1.208955
CDCA5	0.432859	0.037935	0.304641	-1.208029
POLQ	0.436527	0.003754	0.209944	-1.195857
GNG11	0.436677	0.008725	0.231515	-1.19536
LMNB1	0.437591	0.007836	0.222324	-1.192344
ABCC5	0.438048	0.003337	0.209944	-1.190838
SNRNP25	0.440723	0.045847	0.318501	-1.182056
AKR1B1	0.440742	0.041883	0.311902	-1.181994
TCHP	0.441011	0.009102	0.232624	-1.181113
ARRDC2	0.44313	0.009332	0.233624	-1.174198
RPP40	0.444511	0.02202	0.269306	-1.169707
GINS1	0.444681	0.005784	0.216866	-1.169156
BCL7A	0.446295	0.025133	0.276966	-1.163931
RFC4	0.446649	0.007777	0.222324	-1.162787
BLM	0.448381	0.015665	0.254853	-1.157202
BRPF1	0.448408	0.028239	0.284251	-1.157117
METTL7A	0.448753	0.027002	0.281608	-1.156007
NDC80	0.451179	0.016437	0.255513	-1.148228
RNF125	0.452756	0.013213	0.249142	-1.143193
CENPU	0.45528	0.031068	0.287819	-1.135175
SMC2	0.455527	0.002759	0.207585	-1.134392
MTFR2	0.455778	0.006188	0.218509	-1.133595
ORC6	0.457348	0.018107	0.261446	-1.128636

PCNA	0.457815	0.033517	0.29245	-1.127165
PBK	0.457863	0.02137	0.267827	-1.127011
CASP1	0.458135	0.032564	0.290693	-1.126156
TTK	0.460105	0.039035	0.307874	-1.119963
CENPP	0.46033	0.02471	0.27644	-1.119261
NCAPH	0.460885	0.023047	0.272839	-1.117521
RPL15	0.462878	0.034326	0.293505	-1.111296
ZNF367	0.463022	0.003465	0.209944	-1.110847
CCDC92	0.463924	0.045882	0.318501	-1.10804
TCF20	0.464171	0.030731	0.287819	-1.107273
CHEK1	0.464434	0.015966	0.255239	-1.106454
TRAIP	0.464575	0.036094	0.298141	-1.106016
CCNA2	0.465152	0.01011	0.236617	-1.104225
CHAF1B	0.466998	0.029004	0.284251	-1.098513
MCM8	0.472833	0.021288	0.267827	-1.080598
NIPSNAP3A	0.476332	0.002995	0.209944	-1.069961
KIAA1524	0.477721	0.024897	0.276966	-1.06576
CDC25A	0.480863	0.003843	0.209944	-1.056301
RANBP1	0.481377	0.025296	0.277815	-1.054761
NUB1	0.481717	0.006337	0.218509	-1.053741
NASP	0.482525	0.017023	0.258654	-1.051323
ABHD15	0.483001	0.028654	0.284251	-1.049903
C1orf112	0.484303	0.017558	0.26097	-1.046019
ZNF746	0.484565	0.012437	0.247502	-1.045239
MELK	0.485562	0.016846	0.257279	-1.042273
TACC3	0.486959	0.018378	0.262168	-1.038128
TCF20	0.488082	0.046588	0.319763	-1.034803
TCF20	0.488194	0.046818	0.320145	-1.034473
SGF29	0.489553	0.015503	0.254526	-1.030464
CEP55	0.489873	0.002387	0.202854	-1.02952
CDCA8	0.491709	0.039141	0.308321	-1.024124
ERCC6L	0.492478	0.006488	0.218509	-1.021868
GALNS	0.493213	0.001768	0.194485	-1.019718
STIL	0.494871	0.00229	0.202854	-1.014875
MSH2	0.495026	0.003622	0.209944	-1.014424
PIK3IP1	0.495918	0.044217	0.31587	-1.011825
ZNF93	0.496047	0.022682	0.271763	-1.011451
DESI2	0.496273	0.015646	0.254853	-1.010795
KMO	0.496439	0.016718	0.256155	-1.010311
PRKAG2	0.49877	0.022576	0.271582	-1.003553
