# Supplementary material for: Identification of Host Biomarkers of Epstein-Barr Virus Latency IIb and Latency III
Source: mBio. 2019 Jul 2;10(4):e01006-19. doi: 10.1128/mBio.01006-19 (PMC6606803; doi:10.1128/mBio.01006-19)
Supplement: TABLE S2 [file mBio.01006-19-st002.pdf]

Supplamentary Table 2 - Genes Upregulated from Latency IIb to ICAM-1lo LCLs

gene name	fc	pval	qval	log2FC
MGST1	49.72544	0.000849	0.194485	5.635912
PLOD2	42.24167	0.014139	0.250004	5.400595
DST	23.2534	0.000937	0.194485	4.53937
DSG2	19.86327	0.004271	0.213018	4.312031
NRN1	12.01334	0.006612	0.218509	3.586565
DAPK1	10.487	0.017738	0.261004	3.39053
PFKFB4	10.16672	0.024251	0.274864	3.345783
SOX9	9.950645	0.023591	0.27311	3.31479
LINC01055	9.565381	0.02343	0.27311	3.257822
RNF157	9.509785	0.003843	0.209944	3.249413
ROBO1	8.572676	0.04398	0.31587	3.099746
RASSF4	8.462934	0.007568	0.221649	3.081158
ALDOC	8.419223	0.010415	0.236821	3.073687
BCHE	8.282613	0.022078	0.269306	3.050086
CYBRD1	8.235479	0.010617	0.236821	3.041853
LRP12	8.111446	0.004437	0.213018	3.019959
P3H2	8.055228	0.005991	0.216997	3.009925
B4GALT6	7.869454	0.01302	0.249142	2.976264
KIF21A	7.57588	0.002338	0.202854	2.921414
PIEZO2	7.510281	0.033225	0.292267	2.908867
CXXC4	7.318397	0.002695	0.207585	2.871528
EGLN3	7.099019	0.012066	0.245208	2.82762
KCNK1	7.062333	0.035593	0.297164	2.820145
HSPA4L	6.915046	0.00987	0.235682	2.789739
DLGAP1	6.869157	0.008736	0.231515	2.780133
TMOD1	6.419287	0.006173	0.218509	2.682413
BNIP3	6.398666	0.029919	0.28608	2.677771
MSC	6.318902	0.028568	0.284251	2.659674
FBN1	6.236668	0.04183	0.311902	2.640775
SPARC	6.22016	0.009681	0.235276	2.636952
TNFRSF21	5.807535	0.031269	0.287819	2.537926
CDKN1A	5.41234	0.000466	0.194485	2.436252
GOLM1	5.336989	0.018648	0.262359	2.416026
SLC1A1	5.325162	0.017446	0.26097	2.412826
FGF11	5.306269	0.031366	0.287819	2.407698
PLA2G4A	5.280392	0.041261	0.311902	2.400645
OLMALINC	5.262968	0.00559	0.216866	2.395877
C2orf88	5.249814	0.001696	0.194485	2.392266
GADD45A	5.125465	0.002192	0.202854	2.357683

IL2RB	5.10403	0.008689	0.231341	2.351637
KCND2	4.959998	0.025046	0.276966	2.31034
MAP1B	4.833018	0.031102	0.287819	2.272924
MALAT1	4.763036	0.025478	0.277835	2.251881
VEGFA	4.745637	0.010591	0.236821	2.246602
TIMP1	4.718623	0.016575	0.255513	2.238366
CTTN	4.714666	0.016138	0.255513	2.237156
BHLHE40	4.707529	0.00719	0.218509	2.23497
BAIAP2L1	4.697814	0.025002	0.276966	2.23199
RHOB	4.599575	0.021318	0.267827	2.201501
BNIP3L	4.582002	0.023175	0.272839	2.195978
YES1	4.505149	0.002867	0.207585	2.171575
C1019278	4.488334	0.045586	0.317946	2.16618
SOX4	4.437369	0.018201	0.261545	2.149705
NCKAP1	4.41873	0.0205	0.265408	2.143632
ARHGEF25	4.401862	0.004427	0.213018	2.138114
SGCE	4.371886	0.005949	0.216956	2.128256
CMTM3	4.294331	0.01922	0.263611	2.102433
TSC22D3	4.287609	0.016227	0.255513	2.100173
ZNF503	4.273104	0.044865	0.316693	2.095284
ME1	4.256431	0.006931	0.218509	2.089644
MB21D2	4.167388	0.042668	0.313285	2.059143
SHROOM3	4.109297	0.006473	0.218509	2.038892
CST3	4.085622	0.001329	0.194485	2.030556
ACTA2	4.029264	0.046575	0.319763	2.010516
EBI3	3.959438	0.007066	0.218509	1.985296
MIR210HG	3.934008	0.012892	0.248379	1.976
HNRNPLL	3.932329	0.040491	0.310749	1.975384
LOC613266	3.868284	0.006687	0.218509	1.951694
ETV5	3.84247	0.025389	0.277835	1.942034
STC2	3.736423	0.041896	0.311902	1.901658
ZNF697	3.711572	0.034005	0.293505	1.89203
SLC16A3	3.655793	0.018791	0.262359	1.870184
WWTR1	3.636728	0.001916	0.194485	1.862641
MIR210HG	3.636681	0.01796	0.261004	1.862623
GLIS3	3.621278	0.041512	0.311902	1.856499
LGALS1	3.571237	0.048586	0.323155	1.836424
MIR34AHG	3.56267	0.033117	0.291867	1.832959
PTGR1	3.521201	0.047615	0.321385	1.816068
KCNN4	3.492831	0.027228	0.282024	1.804397
MYH10	3.471275	0.018557	0.262359	1.795466
MGLL	3.460183	0.010862	0.238299	1.790848
ARMCX2	3.450081	0.005655	0.216866	1.78663
COL4A5	3.448428	0.002288	0.202854	1.785939
LGALS17A	3.426595	0.029571	0.285409	1.776776

WNT5B	3.377626	0.022995	0.272839	1.75601
TBC1D4	3.367389	0.028894	0.284251	1.75163
SEMA5A	3.28423	5.65E-05	0.194485	1.715555
UGGT2	3.273963	0.001555	0.194485	1.711038
ACTN1	3.271084	0.026545	0.279989	1.709769
SEMA4C	3.262726	0.013434	0.249142	1.706078
ADAM22	3.233244	0.0285	0.284251	1.692982
CXCL14	3.222729	0.005837	0.216866	1.688283
SH3D21	3.189992	0.011413	0.242207	1.673553
NRCAM	3.175645	0.023202	0.272839	1.667049
TOM1L1	3.175523	0.002487	0.203219	1.666994
ARHGEF11	3.151205	0.027338	0.282024	1.655904
RGS2	3.143882	0.039989	0.310042	1.652547
TTLL7	3.122744	0.013972	0.249368	1.642814
LEF1	3.112919	0.008649	0.231211	1.638268
CCDC144B	3.107412	0.047813	0.321716	1.635714
TMTC2	3.090781	0.01074	0.237567	1.627972
MIR3190	3.076174	0.003941	0.209944	1.621137
FSCN1	3.062604	0.01653	0.255513	1.614759
HILPDA	3.052156	0.029829	0.28607	1.609829
MSMO1	3.04978	0.039595	0.309279	1.608705
SERPINH1	3.040818	0.008147	0.225253	1.60446
CLIC6	3.027096	0.025961	0.279308	1.597935
FAM171B	3.008097	0.011772	0.243855	1.588851
CAMSAP2	2.983259	0.01843	0.262168	1.576889
BMP2	2.960566	0.02	0.263838	1.565873
LRIG1	2.959041	0.018086	0.261446	1.56513
PGK1	2.896126	0.031789	0.288638	1.534124
ADAMTS12	2.850658	0.0163	0.255513	1.511295
ZMAT3	2.841982	0.031574	0.288638	1.506898
PYGL	2.838139	0.001883	0.194485	1.504945
HOXB7	2.831382	0.008984	0.232034	1.501506
NID1	2.829115	0.011957	0.244367	1.500351
BCAR1	2.822486	0.012681	0.247556	1.496966
MYLIP	2.807002	0.011062	0.239942	1.48903
ARHGEF17	2.804659	0.031458	0.287852	1.487825
FADS1	2.780854	0.039903	0.309789	1.475528
TCN2	2.770531	0.018153	0.261518	1.470162
LOC647859	2.743314	0.044143	0.31587	1.45592
ACSS2	2.716562	0.024486	0.275506	1.441782
STK3	2.702878	0.005319	0.216866	1.434497
ADCY6	2.699112	0.028048	0.284119	1.432485
FAM162A	2.682268	0.005702	0.216866	1.423454
RHOV	2.673307	0.026606	0.279989	1.418626
CPEB2	2.662466	0.016733	0.256155	1.412763

RAPGEF5	2.659624	0.049495	0.32395	1.411222
MLLT3	2.651341	0.012738	0.247612	1.406722
SIX3	2.609864	0.001159	0.194485	1.383975
TPST1	2.593295	0.01197	0.244367	1.374786
FHL2	2.584186	0.018476	0.262168	1.36971
KIF3A	2.579001	0.009915	0.235796	1.366812
THEMIS2	2.551716	0.025007	0.276966	1.351468
WBP5	2.536723	0.024956	0.276966	1.342966
CD58	2.535663	0.032506	0.290672	1.342363
CTH	2.511614	0.033902	0.293505	1.328615
MAGEF1	2.505505	0.019637	0.263611	1.325102
TRPC1	2.486875	0.013475	0.249142	1.314334
KDM3A	2.485689	0.002012	0.194485	1.313646
MLLT11	2.483973	0.046206	0.318943	1.312649
CABP1	2.454202	0.028339	0.284251	1.295254
KYNU	2.440333	0.013856	0.249142	1.287078
CYB5R2	2.437312	0.03073	0.287819	1.285291
HOXC4	2.425737	0.043701	0.31586	1.278423
SCAMP5	2.41624	0.029802	0.286023	1.272764
CCDC134	2.399311	0.04222	0.313169	1.26262
NDFIP2	2.396012	0.044402	0.315983	1.260635
IRF4	2.394604	0.001822	0.194485	1.259787
DHCR7	2.382114	0.029861	0.28608	1.252243
EPHX2	2.376337	0.022086	0.269306	1.24874
SPAG4	2.371341	0.019473	0.263611	1.245703
CT45A5	2.351417	0.015839	0.255239	1.23353
CRIM1	2.341034	0.004511	0.213018	1.227146
MIR181A2H	2.319769	0.032403	0.290147	1.213981
NR3C1	2.312867	0.012148	0.245453	1.209682
ICAM1	2.311229	0.028747	0.284251	1.20866
SYT11	2.310588	0.018235	0.261545	1.20826
NOL3	2.295768	0.006878	0.218509	1.198977
RUFY4	2.281167	0.025719	0.278206	1.189772
NFKBIA	2.249184	0.020727	0.266878	1.169402
CTTNBP2NL	2.235616	0.002838	0.207585	1.160672
MAGED1	2.228292	0.012704	0.247556	1.155938
EPN2	2.225111	0.004782	0.215764	1.153878
ARHGAP18	2.217892	0.030923	0.287819	1.149189
ACSL1	2.210219	0.028983	0.284251	1.144189
ELOVL6	2.207879	0.021062	0.267819	1.142661
TJP2	2.200473	0.016264	0.255513	1.137814
PPME1	2.19271	0.013851	0.249142	1.132715
ADPRH	2.177485	0.005292	0.216866	1.122663
GBE1	2.173762	0.000308	0.194485	1.120194
LDHA	2.167038	0.020376	0.265408	1.115724

TTC38	2.163231	0.023057	0.272839	1.113187
ENO1	2.145606	0.004652	0.213123	1.101385
LINC00461	2.144731	0.037334	0.302708	1.100797
SREBF1	2.128202	0.040982	0.311042	1.089635
STARD4	2.123843	0.048491	0.322759	1.086677
BCO1	2.111828	0.042708	0.313285	1.078492
GFPT1	2.081307	0.01646	0.255513	1.05749
PGAM4	2.072533	0.022068	0.269306	1.051395
MIR29C	2.06714	0.038659	0.307141	1.047636
CAB39	2.062072	0.046051	0.318692	1.044095
HMGCR	2.05888	0.007812	0.222324	1.04186
TMEM106B	2.056533	0.033416	0.29245	1.040214
ATP7A	2.04594	0.043772	0.31587	1.032764
CPD	2.044924	0.003743	0.209944	1.032047
KCTD1	2.029956	0.019048	0.263611	1.021448
RNF207	2.014935	0.002204	0.202854	1.010733
TMEM170B	2.013117	0.022026	0.269306	1.009431
CMAHP	2.007898	0.046099	0.318762	1.005686
