# Supplementary material for: Identification of Host Biomarkers of Epstein-Barr Virus Latency IIb and Latency III
Source: mBio. 2019 Jul 2;10(4):e01006-19. doi: 10.1128/mBio.01006-19 (PMC6606803; doi:10.1128/mBio.01006-19)
Supplement: TABLE S3 [file mBio.01006-19-st003.pdf]

Supplemental Table 3 - Latency IIb and Latency III Specific Genes

Latency IIb Specific	Latency III Specific
C16orf74	NID1
OSBPL10	MSC
SLA2	COL4A5
RAVER2	GOLM1
ARHGAP42	SERPINH1
SIGIRR	SOX4
GALNT3	NRN1
HEBP1	MGLL
KLHL14	FAM171B
TGFBR2	GADD45A
ARRDC2	UGGT2
KCNK6	GBE1
CCR6	FSCN1
OSBPL8	PPME1
MYO1F	LGALS1
NUGGC	PTGR1
MED13L	RHOB
KDM7A	ELOVL6
HHEX	TOM1L1
SETD7	ICAM1
LYZ	CAMSAP2
JAM3	ACSL1
ZBED2	TNFRSF21
RNF125	TSC22D3
APOBR	SLC16A3
KCNC3	DSG2
DOPEY2	BHLHE40
KMO	ADCY6
RNF130	MGST1
TSPAN13	DST
CCL25	CD58
WHAMMP1	LRP12
FAM46C	NR3C1
RASGEF1B	CYB5R2
IMPA2	ENO1
FCER1G	MB21D2
CSGALNACT1	MAP1B
CHST15	SOX9
FAM177B	LINC01055
RASA2	TMOD1
SERPINB6	WWTR1

CPT1A	KIF3A
PMEPA1	CDKN1A
PWWP2B	ETV5
ZNF860	DLGAP1
BACH2	KIF21A
TBC1D9	B4GALT6
RASGRP2	PGAM4
JAZF1	BNIP3L
UGT8	LDHA
FCRL3	HILPDA
ARL4C	TJP2
C1orf220	EGLN3
NDFIP1	STK3
ZNF682	KCND2
GPD1L	STC2
ELL3	ARHGEF25
TRIB2	SGCE
ADD2	KCNK1
SATB1	NFKBIA
FCRL4	NDFIP2
ANKLE1	RHOV
RBL1	TPST1
BZW2	ADAM22
METTL7A	RNF157
ABHD15	WNT5B
RBMS1	C2orf88
GAPT	MLLT11
S100A10	DHCR7
PDE3B	VEGFA
PTPRO	HOXB7
PIK3IP1	CTTNBP2NL
HIP1R	CYBRD1
REL	TCN2
OGFRL1	ME1
CD24	FBN1
SPOCK2	TIMP1
ZNF486	KCNN4
IFNLR1	PLOD2
WASF1	THEMIS2
ZSCAN18	IL2RB
CLSPN	CTTN
TYROBP	ZNF503
TCHP	EPHX2
LMNB1	ZMAT3
NUDT1	MIR210HG

PPP3CA	TMTC2
TRPM2	NCKAP1
STAC3	BAIAP2L1
PM20D2	EBI3
ALDH16A1	BCHE
FAM111B	SYT11
AKR1B1	HSPA4L
VPS37B	TMEM170B
COQ2	RASSF4
TIMELESS	KYNU
LINGO3	OLMALINC
MLXIP	LOC101927811
PDE4DIP	KDM3A
TAGAP	MLLT3
MILR1	HMGCR
CCDC109B	TBC1D4
CDKN2D	GFPT1
DLGAP1-AS1	MYLIP
NCF4	WBP5
DEF8	PYGL
CNN2	ALDOC
KIAA0226L	SLC1A1
TAF4B	CT45A5
DPEP2	CST3
ORAI2	MSMO1
MTM1	NOL3
SESN3	MIR29C
MIR631	BNIP3
TMEM65	FAM162A
KLF2	PLA2G4A
BIK	FHL2
ARHGAP9	HNRNPLL
CCR1	ROBO1
LINC00674	MYH10
SHC1	BCAR1
ABCA7	TRPC1
PTPRJ	ACTA2
SRGAP2	PFKFB4
USP6NL	LRIG1
ANXA2R	ACTN1
SNORA26	DAPK1
KLHL5	SEMA4C
TTC39C	PGK1
AP1S2	MSRB1
PDCD4	XCL1

GLIPR2	DHCR24
COL4A4	PFKP
TNFSF11	PVRL1
ST3GAL1	ZDHHC18
SNHG5	LOC285628
KIAA0430	SLC12A7
SESN1	GUCY1A3
APPL2	PRKCDBP
QRSL1	LOC400043
CLINT1	TSPAN15
REEP5	ENPP4
SMCHD1	MKNK2
POLE3	TMEM9
PRKCI	PHLDA3
MZB1	TMEM51
FLI1	AQP9
RNF166	HEPH
ADI1	EPDR1
SH2B2	C6orf223
TTLL1	DUSP3
GPR34	DDIT4
HSD17B11	CACNB4
EVI2B	FASN
AMOT	EPS8
RNASEH2B	CCL22
GPR18	GPR153
NRIP1	INPP4B
ESR2	PKN3
SLC25A20	TFB1M
RALGPS2	MYRF
GCNT1	FNIP2
LOC653653	PRICKLE3
BLK	FAM57A
ABTB1	BLVRA
PRNP	SERPINB9
COL4A3	DUSP4
CHST12	MYO6
KAT2B	TIAM2
RASSF1-AS1	EVC2
ATP2A3	MYO1C
YPEL2	ATP1B1
EAF2	FAS
MGAT4A	LOC100996583
GNG7	FAM46A
NLRC3	LGALS3

MAPRE2	CYS1
NADK2	DCBLD1
LBR	JUNB
SLC25A42	PLXNA1
CARD8-AS1	TNFRSF11B
	ACAT2
	DUSP2
	NCF2
	LINC00515
	TNKS1BP1
	HK2
	GPI
	CDC42BPA
	BCL2L1
	HPRT1
	SPATA18
	MTFP1
	CLEC17A
	CRYZ
	PMAIP1
	IER5
	HABP4
	PLD1
	SGMS2
	MIR155HG
	EXTL2
	LHFP
	CAMK4
	OXER1
	ADIRF
	KIAA1549L
	ATP1A1
	GUCY1B3
	BHLHE22
	SERINC2
	RRAD
	VCAM1
	PLA1A
	SLC16A9
	YBX3P1
	HNF1B
	SLC45A3
	PDGFD
	SDC4
	PGM1

GCAT
SPR
CCND2
SCD
PEX11A
ETV4
RAB15
IDH1
ENO2
YBX3
SQLE
RAB9A
ENC1
TSPAN33
CLIP2
MUC13
CCND1
PYCR1
GRHPR
MVD
TCF7
NR6A1
SCCPDH
SIK2
NINJ1
IGFBP4
SYNPO
SRGN
CLIC2
CYB5A
KCNK5
RCN1
NME3
FAM127A
TCEAL4
GEM
TNFRSF8
PTGIR
TPI1P3
GATM
SESN2
ALDOA
CD86
KIF1B
ZNF292

FAM160A1
ZNF267
FOSL1
TRIP10
NPC1
SAMD5
MNX1
CCR7
RGS1
ZNRF1
PSTPIP2
PNKD
BSG
DAAM2
FAM174B
ANKRD37
